# Internet use and health among older adults in China: Indirect associations through social participation and physical activity

**DOI:** 10.1371/journal.pone.0357568

**Published:** 2026-09-21

**Authors:** Haolin Wang, Liang Mu

**Affiliations:** 1 School of Leisure and Social Sports, Jilin Sport University, Changchun, Jilin, China; 2 Winter Olympics School, Harbin Sport University, Harbin, Heilongjiang, China; Ningbo University, CHINA

## Abstract

**Background:**

With the rapid diffusion of digital technologies in later life, Internet use may become an important factor associated with older adults’ health. This study investigated the associations between Internet use and physical and mental health among Chinese older adults, with particular attention to indirect associations through social participation and physical activity.

**Methods:**

Data were obtained from the 2023 Chinese General Social Survey. The analytical sample consisted of 2,219 respondents aged 60 years and above. Descriptive analysis, Pearson correlation analysis, multiple linear regression, bootstrap analysis of indirect associations, and urban–rural subgroup analysis were used to examine the associations among Internet use, social participation, physical activity, and health outcomes.

**Results:**

Internet use was positively related to physical health (β = 0.144, p < 0.001) and mental health (β = 0.090, p < 0.001). Internet use also showed positive associations with social participation (β = 0.138, p < 0.001) and physical activity (β = 0.151, p < 0.001). After social participation and physical activity were included in the models, the associations between Internet use and physical health (β = 0.099, p < 0.001) and mental health (β = 0.061, p < 0.01) remained significant. Bootstrap analysis indicated statistically significant total indirect associations for physical health (95% CI: [0.0228, 0.0404]) and mental health (95% CI: [0.0089, 0.0184]). Among the estimated indirect associations, the association through physical activity accounted for the largest proportion. The interaction between Internet use and urban status was significant only in the physical health model (β = −0.061, p < 0.05).

**Conclusions:**

Internet use is associated with better physical and mental health among older adults in China. Social participation and physical activity statistically account for part of these associations. The stronger association observed for physical health among rural older adults suggests that the health-related role of Internet use may depend on social and resource contexts.

## Introduction

Population ageing and digital transformation are occurring simultaneously in contemporary China. In this context, the Internet has become increasingly integrated into older adults’ daily routines, including information searching, interpersonal communication, service use, and health-related decision-making [1,2]. For older adults, Internet use may provide access to health knowledge, medical information, social interaction, and daily life support. However, older adults also face disadvantages in digital access, digital skills, and the ability to convert digital resources into practical benefits. When digital resources are unevenly distributed or unequally used, digital exclusion may reinforce existing health inequalities [3,4]. Therefore, examining the association between Internet use and health among older adults is important for understanding how digital participation may contribute to healthy ageing and digital inclusion [5,6].

A growing body of evidence suggests that Internet use is related to multiple aspects of health in later life. Previous studies have linked Internet use to self-rated health, physical health, mental health, subjective well-being, depressive symptoms, and health management capacity [7–10]. These associations may arise because Internet use can help older adults obtain health information, communicate with others, access medical or public services, and manage daily activities more conveniently [11–13].

In the CGSS2023, Internet use is operationalized as the self-reported frequency of Internet use during the previous year. This indicator primarily captures the extent of routine digital contact rather than digital literacy, usage purposes, engagement quality, or the ability to evaluate and apply online information. Therefore, frequent Internet use should not automatically be interpreted as evidence of advanced digital competence or effective health-related digital engagement.

Nevertheless, more frequent Internet use may increase older adults’ opportunities to obtain health-related information, communicate with members of their social networks, access daily services, and encounter exercise-related content. Whether these opportunities are associated with health may depend on older adults’ ability to translate digital contact into meaningful social and health-related engagement. Based on this perspective, the present study treats Internet-use frequency as an indicator of routine digital contact and examines whether social participation and physical activity statistically account for part of its association with physical and mental health. Recent research similarly found that digital literacy was positively associated with quality of life among community-dwelling older adults, partly through community participation and health checkups, highlighting the distinction between basic digital contact and the effective use of digital resources [14]. Clinical measurement research has also conceptualized empowerment among older adults with chronic obstructive pulmonary disease in terms of information seeking, participation in care, self-management, psychosocial capacity, and goal attainment, illustrating that effective health-related engagement involves more than access frequency alone [15].

Social participation is one theoretically plausible social correlate that may help account for the association between Internet use and health. It reflects older adults’ involvement in social relationships, daily interaction, emotional exchange, and role maintenance. Research on social relationships and health has long emphasized that social ties and social support are related to health through emotional resources, information exchange, behavioral regulation, and social integration [16,17]. In later life, retirement, reduced mobility, and changes in family or community networks may weaken opportunities for social engagement. Lower social participation may further coincide with loneliness, depressive symptoms, and cognitive decline [18,19]. Digital technologies may partly offset these disadvantages by providing additional channels for communication and social connection. Prior studies have shown that Internet use is associated with broader social networks, social capital, and opportunities for social participation among older adults [20–22]. Accordingly, this study considers social participation as a theoretically plausible social correlate that may statistically account for part of the association between Internet use and physical and mental health.

Physical activity may constitute another theoretically plausible behavioral correlate in the association between Internet use and health. Regular physical activity is essential for maintaining functional ability, reducing chronic disease risk, improving psychological status, and supporting cognitive health in later life [23,24]. Several processes may help explain the association between Internet use and physical activity. More frequent Internet use may increase older adults’ exposure to exercise-related knowledge, health guidance, and self-management information. Digital platforms and wearable devices may also support activity monitoring, feedback, and goal setting. In addition, online communication and peer interaction may provide companionship, comparison, and social motivation that are associated with exercise participation among older adults [25,26]. A multicenter study further found that healthier lifestyles were associated with stronger motivation to participate in physical activity among older adults, highlighting the importance of motivational and social contexts for activity engagement [27]. Existing empirical studies have reported positive associations between Internet use and physical activity among middle-aged and older adults, while digital health interventions have shown potential for improving physical activity and physical function in community settings [28–31]. Accordingly, physical activity is examined as a hypothesized indirect correlate that may statistically account for part of the association between Internet use and health.

Social participation and physical activity may also be statistically connected within a theoretically specified indirect sequence. From a social ecological perspective, individual health behavior is associated not only with personal characteristics, but also with interpersonal relationships, community contexts, and broader social environments [32]. Among older adults, physical activity often occurs in social settings, including neighborhood exercise, group activities, companionship with acquaintances, and community-based leisure participation [33,34]. A group-based aerobic and resistance exercise program among older adults with heart failure also reported improvements in physiological-psychological adaptation, although evidence from a clinical population should not be directly generalized to community-dwelling older adults [35]. Social support and peer interaction may be associated with both the initiation and maintenance of physical activity [36]. In the context of Internet use, more frequent digital contact may be associated with greater opportunities for communication and social engagement, while higher social participation may coincide with increased access to information, encouragement, companionship, and other resources related to physical activity. Accordingly, the sequence from Internet use to social participation, physical activity, and health is treated as a theory-guided analytical ordering rather than an empirically established temporal progression.

Although existing studies have provided important evidence on Internet use and older adults’ health, several questions remain insufficiently examined. First, many studies have focused on general or single health indicators, while fewer have compared physical health and mental health within the same analytical framework [37–40]. Second, prior research has often examined one explanatory factor at a time, such as social capital, information access, healthcare utilization, or lifestyle behavior. Less attention has been paid to whether social participation and physical activity jointly account for part of the association between Internet use and health [41,42]. Third, the association between Internet use and health may vary across urban and rural contexts because of differences in digital resources, healthcare access, and community service conditions [43–46].

**Fig 1:**
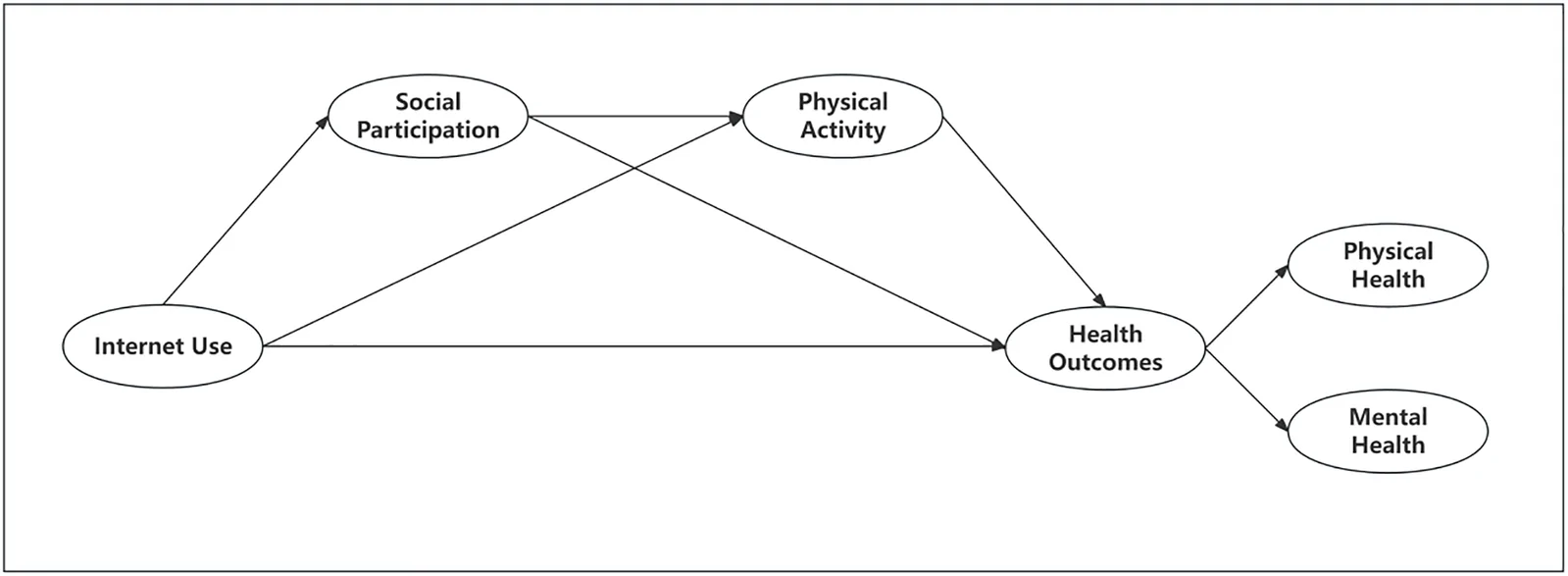
Hypothesized model of Internet use, social participation, physical activity, and older adults’ health. Note: Internet use is the independent variable. Social participation and physical activity are specified as intermediate variables in the statistical models. Physical health and mental health are examined separately as dependent variables. The arrows represent hypothesized statistical associations and should not be interpreted as established causal directions.

To address these gaps, this study uses CGSS2023 data and focuses on Chinese adults aged 60 years and above. It examines the associations of Internet use with physical and mental health, evaluates indirect associations through social participation and physical activity, and further explores whether these associations differ between rural and urban older adults. This study contributes to the literature by distinguishing two health outcomes, considering social and behavioral correlates within the same analytical framework, and examining the contextual role of urban–rural differences. The hypothesized model is presented in Fig 1, and the following hypotheses are proposed.

H1: Internet use is positively associated with both physical and mental health among older adults.
H2: Internet use is indirectly associated with physical and mental health through social participation among older adults.
H3: Internet use is indirectly associated with physical and mental health through physical activity among older adults.
H4: Internet use is indirectly associated with physical and mental health through the theoretically specified sequence of social participation and physical activity among older adults.

## Materials and methods

### Data source

This study used data from the 2023 Chinese General Social Survey (CGSS2023). The CGSS is a large-scale national survey in China that collects information on individuals, households, and social life through a stratified and multistage sampling design. The dataset provides suitable information for examining ageing, social participation, digital behavior, and health-related outcomes.

The CGSS2023 data were accessed for research purposes on 5 April 2026. The de-identified dataset is available to registered researchers upon application through the Chinese National Survey Data Archive (CNSDA). The authors did not have access to any information that could identify individual participants during or after data collection.

The present study restricted the sample to respondents aged 60 years and above. Observations with missing, invalid, or unclear responses on Internet use, social participation, physical activity, physical health, mental health, or key sociodemographic covariates were removed. After sample selection and data cleaning, 2,219 valid cases were included in the final analysis.

### Ethics statement

This study analyzed anonymized secondary data from the 2023 Chinese General Social Survey. The authors were not involved in the original survey implementation and did not have access to personally identifiable information. As the analysis was based only on anonymized public-use data, no additional ethical approval was required for the present study.

### Measures

All variables were constructed from the original CGSS2023 questionnaire items. Before variable construction, non-substantive response codes, including “do not know,” refusal, interview termination, and items not administered in the respondent’s survey mode, were treated as missing. Higher scores were consistently coded to indicate higher levels of the corresponding construct. The exact item wording, original response categories, recoding procedures, final variable ranges, and reliability estimates are reported in S1 Table.

### Dependent variables: Physical health and mental health

The dependent variables in this study were older adults’ health status, measured separately as physical health and mental health.

Physical health was measured using two CGSS2023 items. The first item asked, “How would you rate your current physical health status?” Responses ranged from 1 (“very unhealthy”) to 5 (“very healthy”). The second item asked, “During the past four weeks, how often did health problems affect your work or other daily activities?” Responses ranged from 1 (“always”) to 5 (“never”). Non-substantive responses, including “do not know,” refusal, and interview termination, were treated as missing. Because higher scores on both items represented better health, no reverse coding was required. The mean of the two items was calculated to form the physical health indicator, which ranged from 1 to 5, with higher values indicating better physical health. The inter-item correlation was 0.667, and the Spearman–Brown reliability coefficient was 0.800.

Mental health was measured using four items referring to respondents’ psychological experiences during the previous four weeks. The first item assessed how often respondents felt depressed or downhearted, with responses ranging from 1 (“always”) to 5 (“never”). Because higher scores on this item already indicated better mental health, no reverse coding was required. The other three items assessed how often respondents felt a lack of companionship, felt lonely, and felt left out. These items were originally coded from 1 (“never”) to 5 (“very often”) and were reverse-coded using 6 minus the original score. The mean of the four items was calculated to construct the mental health indicator, which ranged from 1 to 5, with higher scores indicating better mental health. Cronbach’s alpha for the four-item measure was 0.722.

### Independent variable: Internet use

Internet use was measured using the CGSS2023 item asking respondents how frequently they had used the Internet during the previous year, including access through smartphones, computers, tablets, and smart wearable devices. Responses ranged from 1 (“never”) to 5 (“very frequently”). The original coding was retained, with higher scores indicating more frequent Internet use. The final variable ranged from 1 to 5. Because Internet use was measured using a single item, an internal-consistency reliability coefficient was not applicable. This measure reflects the frequency of routine digital contact rather than digital literacy, specific purposes of use, digital skills, or the quality of online engagement.

### Intermediate variables: Social participation and physical activity

Social participation and physical activity were specified as intermediate variables to examine theoretically plausible indirect associations between Internet use and health outcomes.

Social participation was constructed using four items reflecting informal social interaction. The items assessed how frequently respondents met relatives who did not live with them, met friends, engaged in social or recreational activities with neighbors, and engaged in social or recreational activities with other friends. The first two items were originally coded from 1 (“daily”) to 5 (“never”) and were reverse-coded using 6 minus the original score. The latter two items were originally coded from 1 (“almost every day”) to 7 (“never”) and were reverse-coded using 8 minus the original score. The mean of the four recoded items was calculated to construct the social participation indicator. The resulting measure ranged from 1 to 6, with higher scores indicating more frequent social participation. Because the items had different original response ranges, both raw and standardized reliability coefficients were calculated. The raw Cronbach’s alpha was 0.653, and the standardized Cronbach’s alpha was 0.703. This indicator primarily reflects informal everyday social interaction rather than formal organizational, voluntary, or civic participation.

Physical activity was measured using one CGSS2023 item asking how frequently respondents had participated in physical exercise during their leisure time in the previous year. The original responses ranged from 1 (“daily”) to 5 (“never”). The item was reverse-coded using 6 minus the original score so that the final variable ranged from 1 to 5, with higher scores indicating more frequent physical activity. Because physical activity was measured using a single item, an internal-consistency reliability coefficient was not applicable.

### Control variables

The control variables included gender, age, urban–rural status, marital status, and annual personal income. Gender was coded as 1 for women and 0 for men. Age was calculated as 2023 minus the respondent’s birth year and was entered as a continuous variable. Urban–rural status was derived from household registration status. Respondents with non-agricultural household registration or resident household registration previously classified as non-agricultural were coded as urban = 1; respondents with agricultural household registration, resident household registration previously classified as agricultural, or other household-registration status were coded as rural = 0. Marital status was coded as 1 for respondents who were cohabiting, married for the first time, or remarried with a spouse, and 0 for respondents who were never married, separated, divorced, or widowed. Annual personal income referred to respondents’ total personal income in 2022. Because the income variable was strongly right-skewed and included zero values, it was transformed using the natural logarithm of income plus one, ln(income + 1), and entered as a continuous variable. The CGSS2023 questionnaire did not include a direct measure of self-reported physician-diagnosed chronic disease or a standard activities of daily living or instrumental activities of daily living scale. The available item concerning the extent to which health problems affected work or daily activities was incorporated into the physical health outcome and was therefore not additionally entered as a control variable.

### Statistical analysis

Descriptive statistics were first used to summarize the distribution of the main variables. Pearson correlation analysis was then conducted to examine the bivariate associations among Internet use, social participation, physical activity, physical health, and mental health. Multiple linear regression models were estimated to test the association between Internet use and older adults’ health after controlling for sociodemographic characteristics.

In the regression models, physical health and mental health were analyzed separately as dependent variables. Internet use was entered as the core independent variable. Social participation, physical activity, and control variables were then added to the models to observe whether the coefficient of Internet use changed after the intermediate variables were included. The basic regression model was specified as follows:


$$\begin{array}{c} Healt{\text{h}}_{i}=\alpha +\beta_{\text{1}}Internet_{i}+\beta_{\text{2}}Social\;Participation_{i}+\beta_{\text{3}}P\text{h}ysical\;Activity_{i}+\beta_{\text{4}}Controls_{i}+\epsilon_{i} \end{array}$$ (1)

where *Healthᵢ* represents either physical health or mental health for respondent i. *Internetᵢ* refers to Internet use, *SocialParticipationᵢ* refers to social participation, *PhysicalActivityᵢ* refers to physical activity, *Controlsᵢ* represents the set of control variables, and *εᵢ* is the error term.

Internet use and physical activity were measured using five-point ordered response categories, whereas physical health, mental health, and social participation were constructed as mean scores from multiple questionnaire items. In the main analyses, these variables were treated as approximately continuous to preserve the ordering and information contained in the response categories and to facilitate the estimation and interpretation of the indirect-association models. This treatment assumes that adjacent response categories are approximately equally spaced. To assess whether the findings depended on this assumption, two sensitivity analyses were conducted. First, Spearman rank correlations were estimated for the main variables. Second, Internet-use frequency was entered as a categorical variable, with “never” as the reference category, in supplementary regression models that otherwise followed the same specifications as the main regression models. The results are reported in S2 and S3 Tables.

Bootstrap analysis was used to estimate model-specified indirect associations. The number of bootstrap resamples was set to 5,000, and an indirect association was considered statistically significant when its 95% confidence interval did not include zero. The primary indirect-association model specified the sequence “Internet use → social participation → physical activity → health.” This ordering was determined a priori on theoretical grounds rather than selected according to the observed statistical results. From a social ecological perspective, Internet use may be associated with opportunities for communication and social engagement, while social participation may coincide with access to encouragement, companionship, information, and other resources related to physical activity. Physical health and mental health were examined separately.

To assess whether the findings depended on the assumed ordering of the two intermediate variables, an alternative model specifying “Internet use → physical activity → social participation → health” was also estimated. Because the CGSS2023 data are cross-sectional, neither ordering can be interpreted as an empirically established temporal sequence. The estimates represent statistical indirect associations under the specified models rather than evidence of causal mechanisms. Reverse, reciprocal, and common-cause relationships among Internet use, social participation, physical activity, and health remain plausible.

Urban–rural heterogeneity was examined using subgroup regression and interaction models. Rural and urban samples were analyzed separately, and an interaction term between Internet use and urban status was added to the full-sample models. Internet use was mean-centered before constructing the interaction term. All models controlled for gender, age, marital status, and income level.

## Results

### Descriptive statistical results

Table 1 presents the descriptive statistics of the main variables. The final analytical sample included 2,219 respondents aged 60 years and above. The mean score of physical health was 3.18, with a standard deviation of 1.12. The mean score of mental health was 4.25, with a standard deviation of 0.76. The mean value of Internet use was 2.77, with a standard deviation of 1.62. The mean score of social participation was 2.85, with a standard deviation of 1.23, and the mean score of physical activity was 2.52, with a standard deviation of 1.75.

**Table 1:** Descriptive statistics of the main variables.

Variable	N	Mean	Standard deviation
Physical health	2,219	3.18	1.12
Mental health	2,219	4.25	0.76
Internet use	2,219	2.77	1.62
Social participation	2,219	2.85	1.23
Physical activity	2,219	2.52	1.75
Female	2,219	0.53	0.50
Age	2,219	69.94	6.84
Urban	2,219	0.35	0.48
Married/cohabiting	2,219	0.73	0.44
ln(income + 1)	2,219	8.08	3.72

For the control variables, the mean value of female was 0.53, with a standard deviation of 0.50. The mean age was 69.94, with a standard deviation of 6.84. The mean value of urban status was 0.35, with a standard deviation of 0.48. The mean value of married/cohabiting was 0.73, with a standard deviation of 0.44. The mean value of ln(income + 1) was 8.08, with a standard deviation of 3.72.

### Correlation analysis results

Table 2 reports the Pearson correlation coefficients among the main variables. Internet use was significantly and positively correlated with social participation (r = 0.143, p < 0.001), physical activity (r = 0.238, p < 0.001), physical health (r = 0.221, p < 0.001), and mental health (r = 0.156, p < 0.001).

**Table 2:** Correlation matrix of the main variables.

Variables	(1)	(2)	(3)	(4)	(5)
(1) Internet use	1.000				
(2) Social participation	0.143***	1.000			
(3) Physical activity	0.238***	0.140***	1.000		
(4) Physical health	0.221***	0.170***	0.261***	1.000	
(5) Mental health	0.156***	0.087***	0.189***	0.421***	1.000

Note: N = 2,219. Pearson correlation coefficients are reported. *** p < 0.001.

Social participation was significantly and positively correlated with physical activity (r = 0.140, p < 0.001), physical health (r = 0.170, p < 0.001), and mental health (r = 0.087, p < 0.001). Physical activity was significantly and positively correlated with physical health (r = 0.261, p < 0.001) and mental health (r = 0.189, p < 0.001). Physical health was significantly and positively correlated with mental health (r = 0.421, p < 0.001).

### Multiple regression results

Table 3 reports the multiple linear regression results. In Model 1, Internet use was significantly and positively associated with social participation (β = 0.138, SE = 0.017, p < 0.001). The model R² was 0.025, the adjusted R² was 0.023, and the F statistic was 9.574 (p < 0.001).

**Table 3:** Multivariable linear regression associations among Internet use, social participation, physical activity, and health outcomes.

Variables	M1 Social participation	M2 Physical activity	M3 Physical health (total)	M4 Physical health (adjusted)	M5 Mental health (total)	M6 Mental health (adjusted)
Internet use	0.138*** (0.017)	0.151*** (0.024)	0.144*** (0.015)	0.099*** (0.015)	0.090*** (0.010)	0.061** (0.010)
Social participation	—	0.111*** (0.029)	—	0.123*** (0.018)	—	0.059** (0.013)
Physical activity	—	—	—	0.170*** (0.013)	—	0.124*** (0.009)
Age	−0.006 (0.004)	−0.030 (0.005)	−0.043* (0.004)	−0.037† (0.003)	0.041† (0.002)	0.045* (0.002)
Female	0.056* (0.053)	−0.066** (0.072)	−0.074*** (0.047)	−0.071*** (0.046)	−0.035† (0.031)	−0.031 (0.031)
Urban	−0.021 (0.060)	0.189*** (0.082)	0.110*** (0.053)	0.081*** (0.052)	0.141*** (0.036)	0.119*** (0.036)
Married/ cohabiting	−0.020 (0.061)	−0.022 (0.083)	0.007 (0.053)	0.014 (0.052)	0.229*** (0.036)	0.234*** (0.036)
ln(income + 1)	0.042† (0.008)	0.054* (0.010)	0.155*** (0.007)	0.140*** (0.007)	0.103*** (0.005)	0.093*** (0.004)
Observations	2,219	2,219	2,219	2,219	2,219	2,219
R²	0.025	0.114	0.101	0.146	0.112	0.130
Adjusted R²	0.023	0.111	0.098	0.143	0.109	0.127
F	9.574***	40.682***	41.288***	47.142***	46.318***	41.379***

Note: Standardized coefficients (β) are reported; parentheses contain standard errors from the corresponding unstandardized models. All models control for age, gender, urban–rural status, marital status, and ln(income + 1). M2 additionally includes social participation; M4 and M6 additionally include social participation and physical activity. † p < 0.10, * p < 0.05, ** p < 0.01, *** p < 0.001.

In Model 2, Internet use was significantly and positively associated with physical activity (β = 0.151, SE = 0.024, p < 0.001), and social participation was significantly and positively associated with physical activity (β = 0.111, SE = 0.029, p < 0.001). The model R² was 0.114, the adjusted R² was 0.111, and the F statistic was 40.682 (p < 0.001).

In the model estimating the total association with physical health, Internet use was significantly and positively associated with physical health (β = 0.144, SE = 0.015, p < 0.001). The model R² was 0.101, the adjusted R² was 0.098, and the F statistic was 41.288 (p < 0.001). After social participation and physical activity were included, Internet use remained significantly and positively associated with physical health (β = 0.099, SE = 0.015, p < 0.001). Social participation (β = 0.123, SE = 0.018, p < 0.001) and physical activity (β = 0.170, SE = 0.013, p < 0.001) were also significantly and positively associated with physical health. The model R² was 0.146, the adjusted R² was 0.143, and the F statistic was 47.142 (p < 0.001).

In the model estimating the total association with mental health, Internet use was significantly and positively associated with mental health (β = 0.090, SE = 0.010, p < 0.001). The model R² was 0.112, the adjusted R² was 0.109, and the F statistic was 46.318 (p < 0.001). After social participation and physical activity were included, Internet use remained significantly and positively associated with mental health (β = 0.061, SE = 0.010, p < 0.01). Social participation (β = 0.059, SE = 0.013, p < 0.01) and physical activity (β = 0.124, SE = 0.009, p < 0.001) were also significantly and positively associated with mental health. The model R² was 0.130, the adjusted R² was 0.127, and the F statistic was 41.379 (p < 0.001).

### Sensitivity analyses of the treatment of ordered variables

The sensitivity analyses generally supported the robustness of the main findings. As shown in S2 Table, all Spearman rank correlations had the same directions as the corresponding Pearson correlations. Internet use was positively correlated with social participation (ρ = 0.145, p < 0.001), physical activity (ρ = 0.238, p < 0.001), physical health (ρ = 0.218, p < 0.001), and mental health (ρ = 0.165, p < 0.001). The remaining correlations also retained the same directions and significance patterns as those reported in the main analysis.

In S3 Table, Internet-use frequency was treated as a categorical variable, with “never” as the reference category. The Internet-use category indicators were jointly significant in all six supplementary regression models. Compared with respondents who never used the Internet, respondents in the higher-frequency categories, particularly those reporting frequent or very frequent use, generally showed higher levels of social participation, physical activity, physical health, and mental health. However, not every adjacent category contrast was statistically significant, and the pattern should not be interpreted as strictly monotonic. Overall, the results indicate that the main conclusions were not driven solely by treating Internet-use frequency as a continuous variable.

### Bootstrap results for specified indirect associations

Table 4 reports the bootstrap estimates for physical health. The total indirect association between Internet use and physical health was 0.0313, with a Boot SE of 0.0044 and a 95% CI of [0.0228, 0.0404]. The estimated indirect association through social participation was 0.0117, with a Boot SE of 0.0027 and a 95% CI of [0.0069, 0.0175], accounting for 37.38% of the total indirect association. The estimated indirect association through physical activity was 0.0178, with a Boot SE of 0.0034 and a 95% CI of [0.0114, 0.0247], accounting for 56.87% of the total indirect association. The sequential indirect association through social participation and physical activity was 0.0018, with a Boot SE of 0.0005 and a 95% CI of [0.0010, 0.0029], accounting for 5.75% of the total indirect association. The association remaining after the intermediate variables were included was 0.0684, with a Boot SE of 0.0152 and a 95% CI of [0.0386, 0.0981]. The total association was 0.0997, with a Boot SE of 0.0153 and a 95% CI of [0.0697, 0.1296].

**Table 4:** Bootstrap estimates of specified indirect associations between Internet use and physical health.

Specified association	Est.	Boot SE	95% CI	Proportion
Internet use → Social participation → Physical health	0.0117	0.0027	[0.0069, 0.0175]	37.38%
Internet use → Physical activity → Physical health	0.0178	0.0034	[0.0114, 0.0247]	56.87%
Internet use → Social participation → Physical activity → Physical health	0.0018	0.0005	[0.0010, 0.0029]	5.75%
Total indirect association	0.0313	0.0044	[0.0228, 0.0404]	100.00%
Adjusted association	0.0684	0.0152	[0.0386, 0.0981]	—
Total association	0.0997	0.0153	[0.0697, 0.1296]	—

Note: Bootstrap resamples = 5,000. Estimates are unstandardized model-based associations. CI = confidence interval. An indirect association is statistically significant when the 95% CI does not include zero. Proportions refer to the share of the total indirect association.

Table 5 reports the bootstrap estimates for mental health. The total indirect association between Internet use and mental health was 0.0135, with a Boot SE of 0.0025 and a 95% CI of [0.0089, 0.0184]. The estimated indirect association through social participation was 0.0038, with a Boot SE of 0.0015 and a 95% CI of [0.0011, 0.0068], accounting for 28.15% of the total indirect association. The estimated indirect association through physical activity was 0.0088, with a Boot SE of 0.0020 and a 95% CI of [0.0052, 0.0129], accounting for 65.19% of the total indirect association. The sequential indirect association through social participation and physical activity was 0.0009, with a Boot SE of 0.0003 and a 95% CI of [0.0004, 0.0015], accounting for 6.67% of the total indirect association. The association remaining after the intermediate variables were included was 0.0286, with a Boot SE of 0.0104 and a 95% CI of [0.0082, 0.0490]. The total association was 0.0421, with a Boot SE of 0.0103 and a 95% CI of [0.0219, 0.0623].

**Table 5:** Bootstrap estimates of specified indirect associations between Internet use and mental health.

Specified association	Est.	Boot SE	95% CI	Proportion
Internet use → Social participation → Mental health	0.0038	0.0015	[0.0011, 0.0068]	28.15%
Internet use → Physical activity → Mental health	0.0088	0.0020	[0.0052, 0.0129]	65.19%
Internet use → Social participation → Physical activity → Mental health	0.0009	0.0003	[0.0004, 0.0015]	6.67%
Total indirect association	0.0135	0.0025	[0.0089, 0.0184]	100.00%
Adjusted association	0.0286	0.0104	[0.0082, 0.0490]	—
Total association	0.0421	0.0103	[0.0219, 0.0623]	—

Note: Bootstrap resamples = 5,000. Estimates are unstandardized model-based associations. CI = confidence interval. An indirect association is statistically significant when the 95% CI does not include zero. Proportions refer to the share of the total indirect association.

**Fig 2:**
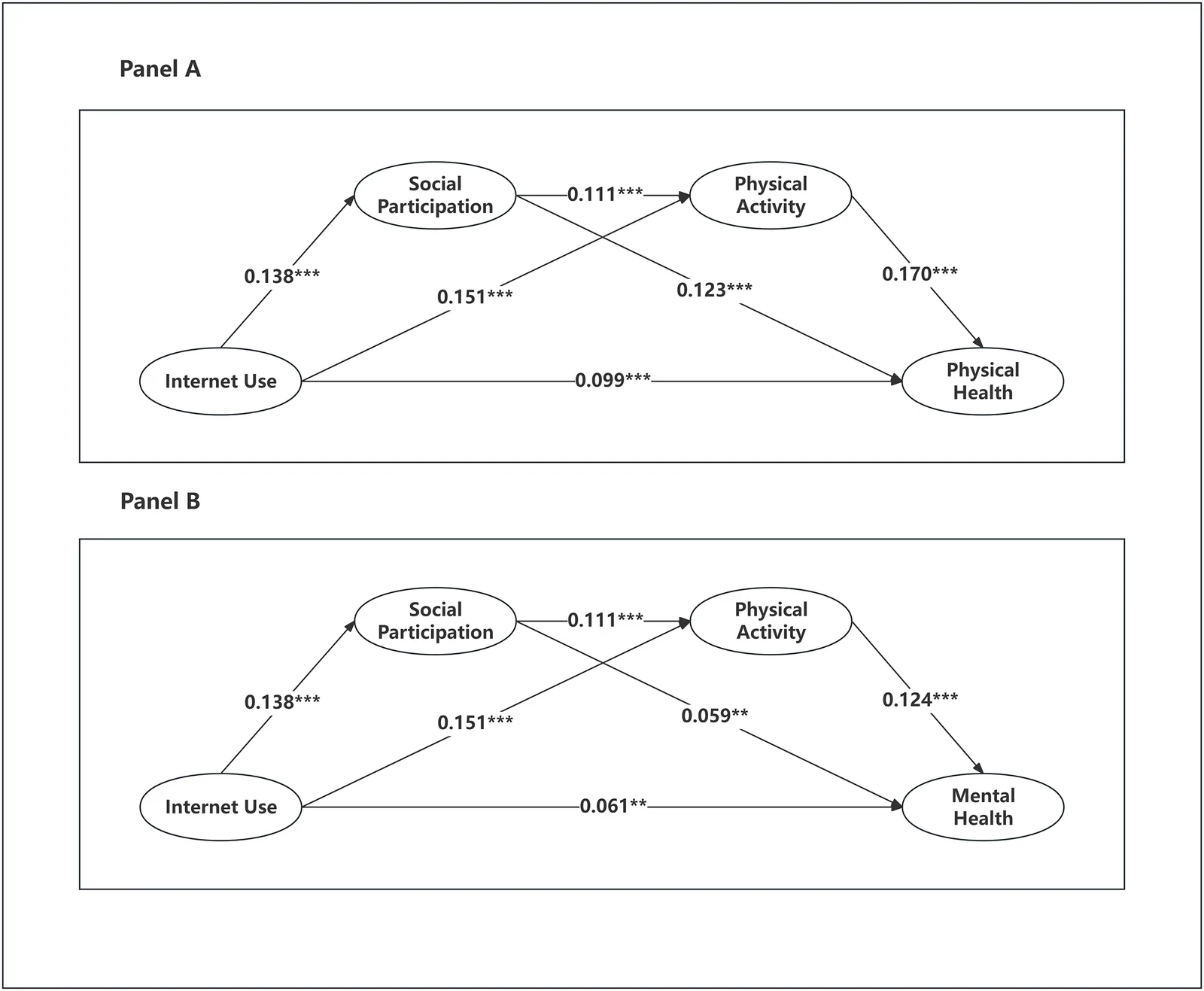
Standardized coefficients from the specified sequential indirect-association models. Panel A presents physical health and Panel B presents mental health as the outcome. Both models include Internet use, social participation, and physical activity and adjust for age, gender, urban–rural status, marital status, and ln(income + 1). Arrows denote model-specified statistical associations and not established causal directions. ** p < 0.01, *** p < 0.001.

The standardized coefficients from the specified sequential indirect-association models are shown in Fig 2.

### Sensitivity analysis using an alternative intermediate-variable sequence

Table 6 reports the bootstrap estimates for the alternative intermediate-variable sequence in the physical health model. Under the specification “Internet use → physical activity → social participation → physical health,” the total indirect association was 0.0313, with a Boot SE of 0.0045 and a 95% CI of [0.0229, 0.0403]. The estimated indirect association through physical activity was 0.0196, with a Boot SE of 0.0035 and a 95% CI of [0.0133, 0.0268], accounting for 62.62% of the total indirect association. The estimated indirect association through social participation was 0.0100, with a Boot SE of 0.0026 and a 95% CI of [0.0054, 0.0156], accounting for 31.95% of the total indirect association. The sequential indirect association through physical activity and social participation was 0.0017, with a Boot SE of 0.0005 and a 95% CI of [0.0009, 0.0028], accounting for 5.43% of the total indirect association.

**Table 6:** Sensitivity analysis of the alternative intermediate-variable sequence for physical health.

Specified association	Est.	Boot SE	95% CI	Proportion
Internet use → Physical activity → Physical health	0.0196	0.0035	[0.0133, 0.0268]	62.62%
Internet use → Social participation → Physical health	0.0100	0.0026	[0.0054, 0.0156]	31.95%
Internet use → Physical activity → Social participation → Physical health	0.0017	0.0005	[0.0009, 0.0028]	5.43%
Total indirect association	0.0313	0.0045	[0.0229, 0.0403]	100.00%
Adjusted association	0.0684	0.0152	[0.0386, 0.0981]	—
Total association	0.0997	0.0153	[0.0697, 0.1296]	—

Note: Bootstrap resamples = 5,000. Estimates are unstandardized model-based associations. CI = confidence interval. An indirect association is statistically significant when the 95% CI does not include zero. Proportions refer to the share of the total indirect association.

Table 7 reports the bootstrap estimates for the alternative intermediate-variable sequence in the mental health model. Under the specification “Internet use → physical activity → social participation → mental health,” the total indirect association was 0.0135, with a Boot SE of 0.0025 and a 95% CI of [0.0089, 0.0187]. The estimated indirect association through physical activity was 0.0097, with a Boot SE of 0.0021 and a 95% CI of [0.0058, 0.0142], accounting for 71.85% of the total indirect association. The estimated indirect association through social participation was 0.0033, with a Boot SE of 0.0013 and a 95% CI of [0.0010, 0.0060], accounting for 24.44% of the total indirect association. The sequential indirect association through physical activity and social participation was 0.0006, with a Boot SE of 0.0002 and a 95% CI of [0.0002, 0.0011], accounting for 4.44% of the total indirect association.

**Table 7:** Sensitivity analysis of the alternative intermediate-variable sequence for mental health.

Specified association	Est.	Boot SE	95% CI	Proportion
Internet use → Physical activity → Mental health	0.0097	0.0021	[0.0058, 0.0142]	71.85%
Internet use → Social participation → Mental health	0.0033	0.0013	[0.0010, 0.0060]	24.44%
Internet use → Physical activity → Social participation → Mental health	0.0006	0.0002	[0.0002, 0.0011]	4.44%
Total indirect association	0.0135	0.0025	[0.0089, 0.0187]	100.00%
Adjusted association	0.0286	0.0104	[0.0082, 0.0490]	—
Total association	0.0421	0.0103	[0.0219, 0.0623]	—

Note: Bootstrap resamples = 5,000. Estimates are unstandardized model-based associations. CI = confidence interval. An indirect association is statistically significant when the 95% CI does not include zero. Proportions refer to the share of the total indirect association.

The alternative intermediate-variable sequence also produced statistically significant indirect associations for both physical and mental health. However, the significance of both model specifications does not establish that either temporal sequence occurred. Instead, the results indicate that social participation and physical activity were statistically interrelated and that the cross-sectional data could not distinguish their temporal ordering. The primary sequence should therefore be interpreted as a theoretically specified analytical model rather than a confirmed behavioral process.

## Urban–rural heterogeneity results

Table 8 reports the subgroup regression results for physical health. In the rural subsample, Internet use was significantly and positively associated with physical health (β = 0.127, SE = 0.019, p < 0.001). Social participation (β = 0.123, SE = 0.023, p < 0.001) and physical activity (β = 0.181, SE = 0.017, p < 0.001) were also significantly and positively associated with physical health. The rural model R² was 0.128, the adjusted R² was 0.124, and the F statistic was 30.328 (p < 0.001). In the urban subsample, Internet use was not significantly associated with physical health (β = 0.036, SE = 0.024, p > 0.05), whereas social participation (β = 0.127, SE = 0.030, p < 0.001) and physical activity (β = 0.141, SE = 0.021, p < 0.001) were significantly and positively associated with physical health. The urban model R² was 0.080, the adjusted R² was 0.072, and the F statistic was 9.461 (p < 0.001).

**Table 8:** Multivariable associations with physical health, stratified by urban–rural status.

Variables	Rural	Urban
Internet use	0.127*** (0.019)	0.036 (0.024)
Social participation	0.123*** (0.023)	0.127*** (0.030)
Physical activity	0.181*** (0.017)	0.141*** (0.021)
Age	−0.014 (0.005)	−0.094* (0.005)
Female	−0.080** (0.058)	−0.040 (0.073)
Married/cohabiting	0.011 (0.068)	0.021 (0.079)
ln(income + 1)	0.149*** (0.008)	0.086* (0.013)
Constant	2.113*** (0.360)	3.495*** (0.438)
Observations	1,454	765
R²	0.128	0.080
Adjusted R²	0.124	0.072
F	30.328***	9.461***

Note: Standardized coefficients (β) are reported; parentheses contain standard errors from the corresponding unstandardized models. All models include Internet use, social participation, physical activity, age, gender, marital status, and ln(income + 1). The constant is unstandardized. * p < 0.05, ** p < 0.01, *** p < 0.001.

Table 9 reports the subgroup regression results for mental health. In the rural subsample, Internet use was not significantly associated with mental health (β = 0.043, SE = 0.014, p > 0.05). Social participation (β = 0.077, SE = 0.016, p < 0.01) and physical activity (β = 0.119, SE = 0.012, p < 0.001) were significantly and positively associated with mental health. The rural model R² was 0.098, the adjusted R² was 0.094, and the F statistic was 22.411 (p < 0.001). In the urban subsample, Internet use was significantly and positively associated with mental health (β = 0.097, SE = 0.016, p < 0.01). Social participation was not significantly associated with mental health (β = 0.017, SE = 0.019, p > 0.05), whereas physical activity was significantly and positively associated with mental health (β = 0.132, SE = 0.014, p < 0.001). The urban model R² was 0.109, the adjusted R² was 0.101, and the F statistic was 13.206 (p < 0.001).

**Table 9:** Multivariable associations with mental health, stratified by urban–rural status.

Variables	Rural	Urban
Internet use	0.043 (0.014)	0.097** (0.016)
Social participation	0.077** (0.016)	0.017 (0.019)
Physical activity	0.119*** (0.012)	0.132*** (0.014)
Age	0.040 (0.003)	0.049 (0.003)
Female	−0.051* (0.041)	0.015 (0.047)
Married/cohabiting	0.225*** (0.048)	0.273*** (0.051)
ln(income + 1)	0.094*** (0.005)	0.055 (0.009)
Constant	3.100*** (0.253)	3.388*** (0.283)
Observations	1,454	765
R²	0.098	0.109
Adjusted R²	0.094	0.101
F	22.411***	13.206***

Note: Standardized coefficients (β) are reported; parentheses contain standard errors from the corresponding unstandardized models. All models include Internet use, social participation, physical activity, age, gender, marital status, and ln(income + 1). The constant is unstandardized. * p < 0.05, ** p < 0.01, *** p < 0.001.

Table 10 reports the regression results for the interaction between Internet use and urban–rural status. In the physical health model, centered Internet use was significantly and positively associated with physical health (β = 0.134, SE = 0.018, p < 0.001), and urban status was significantly and positively associated with physical health (β = 0.089, SE = 0.053, p < 0.001). The interaction between Internet use and urban status was significantly negative (β = −0.061, SE = 0.030, p < 0.05). The model R² was 0.148, the adjusted R² was 0.145, ΔR² was 0.002, and ΔF was 5.698 (p < 0.05). In the mental health model, centered Internet use was significantly and positively associated with mental health (β = 0.053, SE = 0.012, p < 0.05), and urban status was significantly and positively associated with mental health (β = 0.117, SE = 0.036, p < 0.001). The interaction between Internet use and urban status was not statistically significant (β = 0.014, SE = 0.021, p > 0.05). The model R² was 0.130, the adjusted R² was 0.127, ΔR² was 0.000, and ΔF was 0.309 (p > 0.05).

**Table 10:** Interaction between Internet use and urban status in the physical- and mental-health models.

Variables	Physical health	Mental health
Internet use (centered)	0.134*** (0.018)	0.053* (0.012)
Urban	0.089*** (0.053)	0.117*** (0.036)
Internet use × Urban	−0.061* (0.030)	0.014 (0.021)
Social participation	0.124*** (0.018)	0.059** (0.013)
Physical activity	0.172*** (0.013)	0.123*** (0.009)
Age	−0.037† (0.003)	0.045* (0.002)
Female	−0.069*** (0.046)	−0.031 (0.031)
Married/cohabiting	0.013 (0.052)	0.234*** (0.036)
ln(income + 1)	0.140*** (0.007)	0.093*** (0.004)
Observations	2,219	2,219
R²	0.148	0.130
Adjusted R²	0.145	0.127
ΔR²	0.002	0.000
ΔF	5.698*	0.309
F	42.626***	36.804***

Note: Standardized coefficients (β) are reported; parentheses contain standard errors from the corresponding unstandardized models. Both models include social participation, physical activity, age, gender, marital status, and ln(income + 1). Internet use was mean-centered before constructing the interaction term. Urban was coded as 1 = urban and 0 = rural. † p < 0.10, * p < 0.05, ** p < 0.01, *** p < 0.001.

Although Internet use was statistically significant in the urban subsample but not in the rural subsample for mental health, this difference in statistical significance does not itself demonstrate that the two subgroup coefficients are statistically different. The non-significant interaction between Internet use and urban status indicates that there is insufficient evidence of a robust urban–rural difference in the association between Internet use and mental health.

## Discussion

### Internet use is positively associated with physical and mental health among older adults

The findings indicate that Internet use is positively associated with both physical and mental health among older adults, supporting H1. This result is in line with recent studies showing that digital access and Internet function use are related to better health outcomes among Chinese older adults [47]. Evidence from prospective cohort research also suggests that frequent Internet use may be associated with a slower decline in intrinsic capacity among middle-aged and older adults [48].

The interpretation of this association should consider how Internet use was measured. The CGSS2023 indicator captures the frequency of Internet use but does not distinguish whether older adults used the Internet for health information, social communication, entertainment, medical services, or other purposes. It also does not measure digital literacy, the ability to evaluate the reliability of online information, or the capacity to translate digital resources into offline health-related practices. Therefore, the observed association should not be interpreted as evidence that Internet exposure itself improves health. This distinction is consistent with recent evidence linking digital literacy to quality of life through community participation and health engagement [14].

More frequent Internet use may provide greater opportunities to obtain useful information, maintain communication, access services, and encounter health-related content. Telephone-based telenursing has also been reported to reduce physiological and psychosocial stressors among older adults receiving hemodialysis, illustrating one form of digitally supported care while remaining specific to a clinical population [49]. However, whether these opportunities are associated with better health may depend on usage purposes, digital skills, social resources, and community conditions. Moreover, the cross-sectional design prevents causal interpretation, and reverse directionality remains possible because older adults in better health may be more capable of or more willing to use the Internet. The findings should therefore be interpreted as conditional associations rather than causal effects.

### Social participation shows a significant indirect association

The bootstrap results indicated statistically significant indirect associations between Internet use and both physical and mental health through social participation, consistent with H2. This finding is consistent with recent longitudinal evidence showing that Internet usage is associated with fewer depressive symptoms among middle-aged and older Chinese adults, with social participation serving as a partial mediator [50]. Another CGSS-based study also showed that social participation modes, including social and recreational activities and online activities, were significantly associated with older adults’ health status [51].

For older adults, social participation is not only a form of daily interaction, but also a way of maintaining social roles, emotional support, and a sense of belonging. Recent evidence further suggests that social participation is positively associated with older adults’ mental health, although the strength of this association may vary across population groups and types of participation [52]. In the present study, Internet use was positively associated with social participation, and social participation was further associated with both physical and mental health. This suggests that social participation statistically accounted for part of the association between Internet use and older adults’ health.

### Physical activity shows a significant indirect association

The bootstrap results indicated statistically significant indirect associations between Internet use and both physical and mental health through physical activity, consistent with H3. In both health models, the association through physical activity accounted for the largest proportion of the total indirect association. This pattern suggests that physical activity may be an important behavioral correlate in the association between Internet use and older adults’ health.

Physical activity is widely recognized as an important component of healthy ageing. The WHO 2020 physical activity guidelines emphasize that older adults should remain as physically active as their functional ability allows, and regular physical activity is recommended for maintaining health and function in later life [53]. Recent evidence from China has also reported a significant positive association between Internet use and physical exercise among older adults, together with an indirect association through social support [54]. Another study involving middle-aged and older adults reported that social participation may statistically account for part of the association between Internet use and health behaviors, including physical activity [55]. Exercise research among older adults with heart failure has also reported improvements in sleep quality following combined aerobic and resistance exercise, although the clinical context limits generalization to the present population [56].

In the present study, Internet use was positively associated with physical activity, and physical activity was further associated with better physical and mental health. These findings indicate that physical activity statistically accounted for part of the association between Internet use and older adults’ health. The relatively large proportion of this indirect association suggests that physical activity may be particularly relevant when examining the health-related correlates of Internet use.

### Social participation and physical activity show a sequential indirect association

The bootstrap results indicated a statistically significant indirect association from Internet use to health through social participation and physical activity in the specified order, for both physical and mental health. This pattern was consistent with H4. Under the specified model, more frequent Internet use was associated with higher social participation, higher social participation was associated with greater physical activity, and greater physical activity was associated with better health outcomes. These estimates describe associations among variables within the model and do not establish that the variables occurred in this temporal order.

From a social ecological perspective, the specified ordering is theoretically plausible because social interaction and support may coincide with access to information, encouragement, companionship, and other resources related to physical activity [54,55]. However, the supplementary analysis using the alternative intermediate-variable sequence was also statistically significant. This finding suggests that social participation and physical activity are closely interrelated, while the cross-sectional data cannot determine whether one precedes the other, whether their relationship is reciprocal, or whether both are influenced by unmeasured factors.

Accordingly, the sequential indirect association should be interpreted as a theory-guided statistical pattern rather than a confirmed causal chain or behavioral process.

### Urban–rural heterogeneity is mainly reflected in physical health

The results show that the association between Internet use and physical health differed between rural and urban older adults. In the rural subsample, Internet use was significantly associated with physical health, whereas this association was not significant in the urban subsample. The interaction model further showed that the Internet use × urban status interaction was significant only in the physical health model.

The stronger association observed for physical health among rural older adults may reflect differences in the availability of offline health-related resources. Compared with urban communities, rural areas may have fewer healthcare facilities, less convenient transportation, more limited community exercise spaces, and weaker access to professional health information and preventive services. Under these conditions, more frequent Internet use may provide additional opportunities to obtain health information, access service guidance, maintain contact with others, and encounter exercise-related content. Internet use may therefore have a stronger compensatory association with physical health in resource-constrained rural settings [57,58].

By contrast, the urban–rural pattern for mental health was not statistically robust. Although Internet use was statistically significant in the urban subsample but not in the rural subsample, this difference in statistical significance does not by itself demonstrate that the two subgroup coefficients are statistically different. Because the Internet use × urban status interaction was not significant, the subgroup pattern should not be interpreted as evidence of a robust urban–rural difference in the association between Internet use and mental health. Mental health is shaped not only by access to information and services, but also by family relationships, emotional support, loneliness, neighborhood interaction, and community-based social capital. Rural older adults may face distinct constraints related to neighborhood walkability, community infrastructure, population outmigration, and reduced opportunities for sustained social interaction. Previous research has also linked perceived neighborhood conditions and community-based social capital with depressive symptoms among rural older adults [59]. These relational and community-level conditions may not be readily offset by more frequent Internet use alone.

Taken together, the different findings for physical and mental health suggest that the relevance of Internet use may vary across health dimensions and local resource contexts. Physical health may be more closely associated with access to health information, services, and exercise-related resources, whereas mental health may depend on a broader combination of family, social, emotional, and neighborhood conditions. These interpretations remain tentative because the present study did not directly measure community infrastructure, neighborhood walkability, or local social capital.

### Contributions and implications

This study contributes to the literature in three ways. First, it distinguishes physical health from mental health and shows that Internet use is associated with both dimensions, while the strength and urban–rural patterns of these associations vary across health outcomes. Second, it jointly examines social participation and physical activity within the same indirect-association framework and shows that both variables statistically account for part of the associations between Internet use and older adults’ health. Third, it examines urban–rural heterogeneity and finds that the association between Internet use and physical health is stronger among rural older adults.

These findings also have practical relevance, although the implications should be interpreted cautiously given the cross-sectional design. Digital inclusion initiatives for older adults should consider not only Internet access and device ownership, but also digital skills, opportunities for social engagement, and access to appropriate physical activity resources. Randomized evidence from older adults with heart failure also suggests that tele-empowerment can support functional status and health-promoting lifestyles, although this should be interpreted as clinical intervention evidence rather than as evidence about routine Internet use [60]. For rural older adults, stronger coordination among digital infrastructure, primary healthcare services, community support, and digital health education may help create conditions in which digital resources can be used more effectively. Future intervention and longitudinal studies are needed to determine whether such integrated approaches produce measurable improvements in health.

### Limitations and future research

This study has several limitations. First, the use of cross-sectional data limits causal inference. Although bootstrap analyses of indirect associations, an alternative intermediate-variable sequence, and heterogeneity analyses were conducted, the findings should still be interpreted as model-based associations. Future studies could use longitudinal data, panel data, or quasi-experimental designs to better evaluate temporal ordering and potential causal relationships.

Second, Internet use was measured solely by self-reported frequency. This indicator does not capture digital literacy, access quality, device type, purposes of use, depth of engagement, or the ability to evaluate and apply online information. Different forms of Internet use, such as health information seeking, online social interaction, entertainment, online medical consultation, and daily service use, may have different associations with health. Accordingly, the present findings concern the frequency of routine digital contact rather than digital competence or specific forms of digital engagement. Future studies should adopt multidimensional measures of Internet use and digital literacy to clarify which forms of digital engagement are most relevant to older adults’ health.

Third, physical activity was measured mainly by participation frequency, without fully capturing intensity, duration, and specific activity types. Social participation mainly reflected informal everyday interaction rather than formal organizational participation, volunteering, or civic engagement. Future studies should adopt more detailed measures to clarify the specific roles of social participation and physical activity in the observed associations.

Fourth, although this study controlled for several sociodemographic characteristics, residual confounding and omitted variable bias may remain. The CGSS2023 questionnaire did not provide a direct measure of physician-diagnosed chronic disease or a standard ADL/IADL measure. The available item concerning health-related interference with daily activities was already incorporated into the physical health outcome and could not be entered again as an independent control variable. Other unmeasured factors, including family support, digital skills, healthcare accessibility, cognitive status, and regional development, may also be associated with both Internet use and health outcomes. Future studies should use datasets containing richer clinical, functional, family, and community-level covariates.

## Conclusions

This study used data from the 2023 Chinese General Social Survey to examine the associations between Internet use and physical and mental health among older adults in China. Internet use was positively associated with both physical and mental health. Bootstrap analyses identified statistically significant indirect associations through social participation and physical activity, with the indirect association through physical activity accounting for the largest proportion of the total indirect association. A statistically significant indirect association was also observed under the theoretically specified sequence of social participation and physical activity. Given the cross-sectional design, these findings should be interpreted as model-based statistical associations rather than evidence of temporal ordering or causal mechanisms.

The findings further showed that the association between Internet use and physical health was stronger among rural older adults, whereas the urban–rural difference in mental health was not robust. This indicates that the health-related role of Internet use may vary across social and resource contexts. Promoting digital inclusion among older adults should therefore focus not only on Internet access, but also on how digital use can be translated into social participation, physical activity, and tangible health benefits. In particular, more attention should be paid to rural older adults and other groups facing limited health resources, so that digital resources can better support healthy ageing.

## Supporting information

S1 Table Operational definitions, coding procedures, ranges, and reliability of study variables. (DOCX)

S2 Table Spearman rank correlations among the main variables. (DOCX)

S3 Table Sensitivity analyses treating Internet-use frequency as a categorical variable. (DOCX)

S4 File SPSS syntax for variable construction and statistical analyses. (ZIP)
