# Supplementary material for: Internet use and health among older adults in China: Indirect associations through social participation and physical activity
Source: PLoS One. 2026 Sep 21;21(9):e0357568. doi: 10.1371/journal.pone.0357568 (PMC13592573; doi:10.1371/journal.pone.0357568)
Supplement: Supplementary file 1 [file pone.0357568.s001.docx]

**S1 Table. Operational definitions, coding procedures, ranges, and reliability of study variables**

| **Construct** | **CGSS2023 item(s) and original response categories** | **Recoding and computation** | **Final range** | **Reliability** |
| --- | --- | --- | --- | --- |
| Physical health | A15: current physical health, 1 = very unhealthy to 5 = very healthy; A16: frequency with which health problems affected work or daily activities during the previous four weeks, 1 = always to 5 = never. | Mean of A15 and A16; no reverse coding. | 1–5 | Inter-item r = 0.667; Spearman–Brown = 0.800 |
| Mental health | A17: depressed or downhearted, 1 = always to 5 = never; C16a–C16c: lack of companionship, loneliness, and feeling left out, 1 = never to 5 = very often. | C16a–C16c were reverse-coded as 6 − original score; mean of A17 and the three recoded items. | 1–5 | Cronbach’s α = 0.722 |
| Internet use | A28 item 5 (A285): Internet use during the previous year via smartphones, computers, tablets, or smart wearable devices, 1 = never to 5 = very frequently. | Original coding retained. | 1–5 | Single item; not applicable |
| Social participation | A30 items 6–7: gatherings with non-coresident relatives and friends, 1 = daily to 5 = never; A31a–A31b: social or recreational activities with neighbors and other friends, 1 = almost daily to 7 = never. | A30 items were reverse-coded as 6 − original score; A31 items as 8 − original score; mean of the four recoded items. | 1–6 | Raw α = 0.653; standardized α = 0.703 |
| Physical activity | A30 item 9: leisure-time physical exercise during the previous year, 1 = daily to 5 = never. | Reverse-coded as 6 − original score. | 1–5 | Single item; not applicable |
| Gender | A2: 1 = male, 2 = female. | 0 = male; 1 = female. | 0–1 | Not applicable |
| Age | A3a: year of birth. | 2023 − year of birth; entered as a continuous variable. | 60–94 | Not applicable |
| Urban–rural status | A18: household-registration status. | 1 = non-agricultural registration or resident registration previously classified as non-agricultural; 0 = agricultural registration, resident registration previously classified as agricultural, or other registration status. | 0–1 | Not applicable |
| Marital status | A69: 1 = never married, 2 = cohabiting, 3 = first marriage with spouse, 4 = remarried with spouse, 5 = separated, 6 = divorced, 7 = widowed. | 1 = cohabiting, first marriage with spouse, or remarried with spouse; 0 = never married, separated, divorced, or widowed. | 0–1 | Not applicable |
| Annual personal income | A8a: total personal income in 2022. | Natural-log transformation, ln(income + 1); entered as a continuous variable. | 0–16.117 | Not applicable |

Note: Non-substantive response codes were treated as missing before variable construction. α = Cronbach’s alpha. Internal-consistency reliability is not applicable to single-item indicators or sociodemographic variables.
