# Supplementary material for: Internet use and health among older adults in China: Indirect associations through social participation and physical activity
Source: PLoS One. 2026 Sep 21;21(9):e0357568. doi: 10.1371/journal.pone.0357568 (PMC13592573; doi:10.1371/journal.pone.0357568)
Supplement: Supplementary file 2 [file pone.0357568.s002.docx]

**S2 Table. Spearman rank correlations among the main variables**

| **Variables** | **(1)** | **(2)** | **(3)** | **(4)** | **(5)** |
| --- | --- | --- | --- | --- | --- |
| (1) Internet use | 1.000 |  |  |  |  |
| (2) Social participation | 0.145*** | 1.000 |  |  |  |
| (3) Physical activity | 0.238*** | 0.149*** | 1.000 |  |  |
| (4) Physical health | 0.218*** | 0.167*** | 0.267*** | 1.000 |  |
| (5) Mental health | 0.165*** | 0.067** | 0.201*** | 0.439*** | 1.000 |

Note: N = 2,219. Spearman rank correlation coefficients are reported. ** p < 0.01, *** p < 0.001.
