# Supplementary material for: Internet use and health among older adults in China: Indirect associations through social participation and physical activity
Source: PLoS One. 2026 Sep 21;21(9):e0357568. doi: 10.1371/journal.pone.0357568 (PMC13592573; doi:10.1371/journal.pone.0357568)
Supplement: Supplementary file 3 [file pone.0357568.s003.docx]

**S3 Table. Sensitivity analyses treating Internet-use frequency as a categorical variable**

| **Variables** | **M1 Social participation** | **M2 Physical activity** | **M3 Physical health (total association)** | **M4 Physical health (adjusted model)** | **M5 Mental health (total association)** | **M6 Mental health (adjusted model)** |
| --- | --- | --- | --- | --- | --- | --- |
| Internet use = 2 (rarely) | 0.198* (0.086) | −0.077 (0.117) | 0.277*** (0.076) | 0.260*** (0.074) | −0.034 (0.051) | −0.039 (0.051) |
| Internet use = 3 (sometimes) | 0.207* (0.090) | 0.334** (0.122) | 0.311*** (0.079) | 0.248** (0.077) | 0.037 (0.053) | 0.009 (0.053) |
| Internet use = 4 (often) | 0.494*** (0.079) | 0.388*** (0.109) | 0.375*** (0.070) | 0.268*** (0.069) | 0.037 (0.047) | −0.007 (0.047) |
| Internet use = 5 (very frequently) | 0.365*** (0.074) | 0.641*** (0.101) | 0.399*** (0.065) | 0.281*** (0.064) | 0.190*** (0.044) | 0.139** (0.044) |
| Joint F for Internet-use categories | 11.450*** | 13.070*** | 12.612*** | 7.232*** | 6.072*** | 3.869** |
| Observations | 2,219 | 2,219 | 2,219 | 2,219 | 2,219 | 2,219 |
| R² | 0.030 | 0.116 | 0.104 | 0.149 | 0.115 | 0.133 |

Note: Unstandardized coefficients are reported, with standard errors in parentheses. Internet use = 1 (“never”) is the reference category. All models control for age, gender, urban–rural status, marital status, and ln(income + 1). M2 additionally includes social participation. M4 and M6 additionally include social participation and physical activity. The joint F row tests the null hypothesis that all four Internet-use category coefficients equal zero. * p < 0.05, ** p < 0.01, *** p < 0.001.
